# Abstracts &c.

**Published:** 1889-09

**Authors:** 


### ABSTRACTS AND GLEANINGS.

**Methacetin—A New Antipyretic.**—Dr. Franz Hahnert, assistant to Professor von Jaksch, describes in the *Pharmaceutische Post* for April 7, 1889, the most recent addition to our rapidly growing list of synthetic medicinal compounds. This time it is an antipyretic, and in composition might be described under the name of para-acetanisidin, its formula being

$$C_6H_4 \begin{cases} OCH_3 \\ NH.C_2H_3O \end{cases}$$

This substance is a combination of the radical acetyl with anisidine, and is, therefore, an acetyl combination of the methyl ether of para-amedophenol. From the fact that para-acetpheuetidin is commonly described under the name of phenacetin, the precedent is formed of describing this compound under the name of methacetin.

This substance occurs in the form of a powder, formed of slightly reddish scales, without odor; slightly bitter to the taste; soluble in warm water, to a less degree in cold water; while it is very soluble in cold and warm alcohol. It is not more soluble in acid and alkaline liquids than when neutral; its point of fusion is 127° C. From experiments made on animals, it was seen that methacetin, as might be expected from its analogy in combination to phenacetin, possesses the power of reducing temperature,—three, four, or five degrees being lost within a few hours after its administration. Doses of 45 grains given to rabbits are markedly poisonous, producing convulsions in the posterior limbs, then in the anterior members, as in the case of poisoning with antipyrin. Its action is principally exerted on the central nervous system, death being produced in rabbits by administration of only 45 grains. In doses of from 2 to 3 grains, given to children, it exerts a marked antithermic action, the reduction of the temperature being gradually produced, remaining for several hours at the lowest point, and then gradually increasing. Frequently marked perspiration is produced by its use within an hour after its administration. If the administration of the drug corresponds to the day of remission of the fever, the thermal fall is produced more rapidly, and has a longer duration than under other circumstances. The author has employed it in three cases of pulmonary tuberculosis, one of tubercular meningitis, and one of pneumonia, the patients in all the cases being betwen 6 and 13 years of age. In one instance it produced slight collapse, but never vomiting, ringing in the ears, vertigo, or erythema. The urine during the administration of this drug reduces the potasso-cupric test for sugar, but does not itself contain sugar, and there is no hæmoglobinuria. Methacetin, in one per cent. solution, prevents coagulation in milk and ferment. It seems worthy of further study.

**Diarrhœa of Infants, with Green Discharges.**—Give the mother tincture of chamomile, two drops in one-half glass of water, teaspoonful every half-hour or hour.—*Ib.*

**The Oil of Turpentine** would appear to be a remedy always to be tried in cases of membranous croup before resorting to intubation or tracheotomy. Loewentaner reports (*Med. News*) two cases of severe stenosis of larynx, in both of which the administration of a coffeespoonful of turpentine was almost immediately followed by expectoration of the membranes, and subsequent small doses internally or by inhalation led to complete recovery. Several coffeespoonfuls of the oil may be administered during a night and day for several days if the membranes reform.—*Ex.*

**Pruritus Ani.**—A correspondent of *Practice* gives the following which he states is an excellent application in anal pruritus:

| | | |
|---|---|---|
| ℞ | Hydgr. chl. mit | ʒ i |
| | Balsam Peru | ʒ iss |
| | Carb. acid | grs. xx |
| | Lanolin | ℥ i |

M. Ft. ointment.
M. Sig. Apply once or twice daily after sponging with hot water.

**Caffeine in Migraine.**—Dr. E. J. Overend, of Oakland, writes in the Pacific Medical Journal regarding his personal experience with caffeine in sick headache. Having himself been a victim for many years, he has ample opportunity to test the comparative values of many drugs, from the study of which he rises with the conviction that caffeine comes as near being a specific in migraine as does quinine in intermittent fever. He is even inclined to rate its claims to specificity beyond those of the latter, but like that drug, its prophylactic power outranks its curative value. His practice is to anticipate the attack by doses of two to five grains hourly, dispensed in capsules; this seldom fails to abort the attack if taken sufficiently early, or if not so taken, a marked amelioration of the symptoms results. He counsels his patients not to procrastinate, as they are prone to do, but to begin the dosage promptly as soon as the well-recognized prodomes appear. When capsules are objectionable to the patient and a solution is preferred, the salicylate of sodium should be combined with the citrate of caffeine, his belief being that the former salt not only renders the latter more stable, but also acts as a synergist. If recently reported experiments are correct, caffeine has another noteworthy advantage in that it does not enslave as do some of the narcotic remedies that are used in this affection. He has not found it necessary to increase the dose progressively, as is commonly the case with the habit-forming drugs.

Of the various proprietary preparations containing caffeine he has no commendatory evidence to give; they contain too little caffeine and too many unnecessary adjuncts, and of the effervescent compounds he remarks that they seem to contain more "fiz" than caffeine, and give him the impression that cheapness plays a part in their construction. Dr. Overend's paper is written in a scholarly and sympathetic vein, and we recommend it to the many medical men who are victims to migraine, as a useful bit of post-graduate instruction.—*Philadelphia Medical News.*

**Medical Journals.**—Dr. W. W. Dawson, in his presidential address to the American Medical Association, referred thus to the Medical journals:

Medical journals, metropolitan and provincial, are the heralds, the vanguards of medical progress, the exponents of professional culture. They are closely associated with the colleges in education and in post graduate instruction. In them appear the best thoughts of the best men; they constitute the great forum of intellectual combat; upon their pages pretension is analyzed and estimated, and worth recognized; that which is new or original is endorsed, or rather encouraged; it is only the plan, the original investigation which is endorsed; the results, the conclusion must be subject to the crucible of test and trial.—*St. Louis Medical and Surgical Journal.*

**The Mercury Salts** are among the most important of the substances affected by actinic light. The conversion of the *mercurous*, or non-poisonous, to the *mercuric*, or poisonous salts, is likely to be attended with fatal results, when the change has been sufficiently great. Calomel, therefore, should be kept in amber bottles away from light to prevent its being converted into the poisonous corrosive chloride.

**The Elixir of Life.**—At the meeting of the Society of Biology on the 1st of June last, its President, the celebrated Dr. Brown-Sequard, announced a remarkable discovery. He began by speaking of the effect on the nervous system upon glands, and reciprocally, the effect of glands upon the nervous system, independent of their function of removing from the blood products to be excreted, for they may at the same time secrete substances which exert a marked influence over the nervous system and circulation. He had several times tried to graft on one animal parts of another animal containing testicles, and in one case had succeeded. A feeble old dog, upon which he succeeded in grafting in the thigh a piece of testicle, regained his vigor. Then changing his method of procedure, he began trying hypodermic injections of the blood from the testicle, or of a liquid obtained from the triturations of the testicle, or of seminal vesicles. These injections were first tried upon animals; and no harm resulting, he then tried upon his own person injections of a liquid composed of fluid from the triturated testicle and the blood of the spermatic veins of dogs or guinea pigs. These injections caused no other inconvenience than local pain and redness. The general results were, however, remarkable. Muscular power became greatly increased; intestinal atony disappeared, defacation becoming normal and easy, and the bladder recovered its contractile power. The brain, also, recovered its vigor, and the other systems participated in this remarkable return of youth. Dr. Brown-Sequard thinks that this action is chiefly due to the blood of the spermatic vein, for injections of semen or of filtered spermatic liquid cause only abscesses.

At a meeting of the Society, on 15th of June, the doctor again took up the subject, reiterating the wonderful changes the injections had caused in his own person, renewing his physical and mental faculties. He especially called attention to the fact of the permanency of the effects of the injections. It had been 11 days since he took his last, and its good effects still remained. The effects are evidently dynamic and not organic, for one would not conceive what organic modification could he effect in the spinal centers of the sphincters, for example. At the next meeting he intended to speak of this dynamic influence. Castrated women, he believed, were veritable female eunuchs. It would be interesting to try the effects of injections of ovarian liquid upon females, and he would like for the lady physicians to try its effects on their own persons or on other women. He appealed to the scientific press to help him solve this problem.—*Le Progres Med.*, June 8 and 22, 1889.

**The Testicle as a Rejuvenator.**—Twenty years ago, at least, Dr. Brown-Sequard exhibited tendencies towards a belief that the testicle might be of value for other purposes than the impregnation of the ovum, provided it was taken when young—that it was competent, when its vital principles were properly injected for the respective purposes, not only to call into existence the very young, but to rejuvenate the aged. It seems that this idea has continued to germinate in the brain of the learned but eccentric, physiologist all these years. In 1873 he made experiments with grafts of testicular tissue upon dogs, and to his delight succeeded, as he thought, in renewing the youth of one wretched old cur. Since then he has continued these strange investigations at various times, and during the month of June, this year, made two separate communications to the Societe de Biologie of Paris upon this subject, describing the methods used and the supposed results. He, apparently, thinks he has discovered a sort of *elixir vitæ*, or fountain of perpetual youth, of simpler composition than those elixirs so sedulously compounded by the mediæval philosophers, and easier of access than the elusive fountain which enticed poor Ponce de Leon to his fond and fatal journey. According to the reports of Brown-Sequard's communications given by the French journals, he has been experimenting with a fluid obtained by crushing and washing the testicles of young animals, which was mixed with blood from the spermatic veins and water. This fluid he injected into his own subcutaneous cellular tissue almost every day for two weeks, with results so gratifying that he hastened to communicate them to his biological confreres. Notwithstanding his ripe age, between seventy and eighty years, he experienced a rejuvenescence of all his forces, physical and mental. The former healthy and vigorous contractility of the intestines and bladder had returned, as also had his general muscular strength. Intellectual labor had again become easy to him. Dr. Brown-Sequard did not succeed apparently in inoculating his hearers with his own enthusiasm for his procedure. Scepticism and physiological objections found expression through MM. Dumont-

Pallier and Fere. Nor did the Society treat his results with sufficient seriousness to discuss the question as to the value of the ovary for similar purposes, or as to the possible results of injecting subcutaneously an ovarian aqueous extract into the male and testicular extract into the female.—*Boston Medical and Surgical Journal.*

**Creolin as an Antiseptic.**—Creolin is being actively discussed on the continent at present, as well as in this country. Whether it will stand the test of the rigid examinations to which it is being subjected, remains yet to be seen. The main point of discussion is whether creolin is an antiseptic or not. German authorities seem specially interested, and their opinions are quoted at length in many medical journals.

Dr. Eisenberg states that he has found two per cent. to five per cent. solutions of creolin to be powerfully antiseptic, kill almost immediately streptococci, staphylococci, cholera baccilli, and other bacteria. He prophesies a brilliant future for the drug. Seidel and Hornicke agree with Eisenberg, and consider ten per cent. creolin gauze or cotton to be an admirable substitute for bichloride gauze. The former preparations may now be purchased in the German market.

Hunermann, as the result of a long series of investigations states that creolin has no right among antiseptics. It undoubtedly retards and in some cases even prevents the further growth of bacteria, but does not kill them. Koch's experiments have proved that the oil of mustard in week solutions may hinder the growth of bacteria, yet no one would for a moment regard it in the light of an antiseptic, yet this, he claims, is what is being done with creolin.

Dr. Korturn (*Centralbl. f. Gynak.*, No. 6, 1889) and Dr. Bunsen have found it a most admirable antiseptic. They claim that it is not so irritating as carbolic acid, and that it also possesses styptic properties. Korturn uses wet creolin gauze exclusively for plugging the uterine cavity in cases of severe hemorrhage. For washing the vagina, Bunsen uses only a one-half per cent. solution. He states also that a two per cent, solution immediately kills pediculi pubis.—*Medical News.*

**Infantile Diarrhœa.**—The medical treatment is divided to meet the demands of three sets of cases.

1st. Those with vomiting, colic, convulsions, frequent greenish stools and great exhaustion. At the onset give a tablespoonful of the following mixture:

| | | |
|---|---|---|
| ℞ | Ol. ricini | ℥i. |
| | Glycerin | ℥ii- |
| | Ol. cassiæ | gtt. i. |

After it has operated freely, give some antiseptic combined with a small dose of opium. Salicylate of sodium is perhaps the best. For a child two years old the correct formula will be.

℞ Sodii salicylatis grs. iv.
Tr. opii deod. gtts. x.
Syr. simp. ℥ i.
M. Sig. One teaspoonful every three hours.

The result is rapid and satisfactory.

2nd. The diarrhœa may be tolerably frequent and of a vivid grass green color but unattended by vomiting and marked prostration. This variety is rapidly cured by latic acid. A two per cent, solution may be given in doses of one teaspoonful every one or two hours.

3rd. This type is commonly insidious in origin; the stools being softer and more frequent than usual for a long time before the onset of alarming symptoms; or if engrafted on a pre-existing cholera infantum. Fully developed, the stools are green or pale in color, moderately thin, and containing sago like pellets of mucous with here and there specks and streaks of blood. Later on we find shreds and strings of mucous like substance apparently caused be sloughing from superficial ulcers of the colon. Examine carefully into the sanitary surroundings of the patient and eliminate all errors in diet. A moderate dose of castor oil will remove all irritating matter from the intestine. Then a small dose of opium and bismuth subnitrate will quiet the nervous system and soothe the intestinal mucous membrane. As soon as the number of the stools are reduced to a moderate number, small doses of Fowler's solution of arsenic may be added, and the opium gradually discontinued. Bloody stools are frequently corrected by injections of nitrate of silver; one grain to the pint; at intervals of twelve to twenty hours. When convalescence is established a sojourn to the seaside is advisable.—Dr. A. L. Scott, *New Eng. Medical Monthly*, April, 1889.

**Cocaine as a Hæmostatic.**—An anonymus writer in the *Scalpel* on the hæmostatic action of cocaine, remarked that, for the last three years he had recourse to the subcutaneous injection of the hydrochlorate of cocaine to produce local anæsthesia, that after these injections there was no hemorrhage, or at least the flow of blood was less than when he did not employ cocaine. From this fact the idea struck him that it would be a useful means against excessive hemorrhages, which are sometimes difficult and long to arrest. With the view of correcting the flow of blood, the author tried the direct application to the bleeding surface, of pads of charpic imbibed in the following solution: Hydrochlorate of cocaine, 1 gram; alcohol, 5 drops; cherry-laurel water, 5 grams. He sometimes applied the powder of cocaine to the wound, at others he employed a subcutaneous injection of the same substance, in the neighborhood of the seat of hæmorrhage. The first mode of application succeeded in rapidly arresting a severe attack of epistaxis. Suppositories containing from 15 to 20 centigrams of cocaine have always succeeded in arresting persistent oozing of blood. Commenting on this note, Dr. Fano, in the *Journal d' Oculistique*, observed that this latter dose of cocaine is not without danger. It is well known with what facility and rapidity is accomplished the function of absorption in the rectum.—*Journal of the A. M. A.*

**The Utilizing of Condemned Murderers.**—The practical mind of Dr. Frank L. James, of our sprightly, monthly contemporary, would utilize the bodies of such criminals, *ante mortem*, for experimental pathology. This is a good suggestion, and in lieu of the judicial condemnation formulary: "Hanged by the neck until you are dead—dead—dead, and may God have mercy on your soul," we hope to see the time come when, in pronouncing sentence for capital crime, the judge will solemnly say: "And now you are sentenced under the laws you have violated, to pay the righteous penalty of your crime. You will therefore, this day choose the method by which you prefer to die for the benefit of Science and that society you have wronged, that, dying you may serve mankind better than when you lived, and in part at least, make propitiation to the world and to God for your great crime, and may God have mercy on your soul." Let the condemned then choose whether by poison, by inoculation of disease, by vivesection or electricity.

Give the condemned murderer a chance to make some atonement for his crime before he goes hence.

**The Bishop Autopsy.**—Drs. J. A. Irwin, Frank Furguson and H. Hance were arraigned in court on Tuesday, on indictments for misdemeanor, found against them by the Grand Jury for "unlawfully making a dissection of the body of Washington Irving Bishop, on May 13th, without authority of law, and not in pursuance of permission given by the deceased." They pleaded not guilty, and were each held in $500 bail.—*New York Journal.*

It was claimed by the mother and some of the relatives and friends of the diseased mind-reader that he was not dead, but only slept in cataleptic simulation of death.

But Bishop had not the precursory rigidity of catalepsy, and his physicians were too familiar with neuropathic symptomatology to make the mistake even of taking a trance subject for a cadaver.

Bishop's spells during life were syncopal, possibly epileptoid. His fatal illness was epileptic as the autopsy revealed. He stimulated, as Foster did, when he needed sleep and ought to have slept. Antipyrine too in large quantities is said to have been a favorite self-prescribed remedy with him for nervousness and cephalalgia. Bishop was imprudent, wore himself out, and died of the consequences of cerebral exhaustion.—*Alienest and Neurologist.*

[The fact that Bishop the mind reader was subject to cataleptic or trance conditions is confirmatory of the theory advanced by Dr. R. C. Word, in a paper read before the Georgia Medical Society, Apr., 1887, entitled Duality of the Brain, etc.

In that paper it was held that mind reading is a phaze of mesmerism, the mind reader being in the electro negative or positive state, the same as that of a mesmerized party in which state he becomes conscious of the thoughts of the party with whom he becomes in *rapport.* —ED. RECORD.]

**Morphine-Vaselin as a Local Application in Cancer.**—According to Dr. B. W. Richardson, morphine combined with vaselin forms one of the best possible sedative applications in cases of external malignant disease in which there is ulcerative breach of surface with continuous pain. The mode of prescribing the preparation runs as follows:

℞ Vaselin pure ℥ j.

Chloroform, ʒ ij.

Morphine, gr. iv.

Mix thoroughly and make into an ointment.

By using the chloroform a larger quantity of morphine can be introduced than if the alkaloid were used alone, the distribution through the mass being more complete. The chloroform, in the proportions named, causes, as a rule, no irritation, but acts as a sedative. Chloroform also is a powerful antiseptic, and as such is a good remedy in the class of cases under consideration.

Morphine-vaselin is most conveniently applied thinly spread over a piece of fine lint or soft linen. It is easily removable without injury to the surface upon which it is applied, and it may, therefore, be used even on surfaces from which the danger of hæmorrhage is foreseen. The dressing may be renewed twice in the course of twenty-four hours.—*Med. News.*

**Employment of Loboline in the Treatment of Asthma.**—In the Therapeutic Gazette for September 5, 1886, we referred to an article by Dr. Nunes as to the use of lobelia inflata in asthma. Dr. Nunes employed the tincture in doses of ½ to 1 ounce without ever meeting with an accident, and claims that the inertness of this drug in the hands of others is owing to the insufficient dose employed. At the recent Brazilian Congress of Medicine and Surgery, held at Rio Janeira, Dr. Silva Nunes presented a subsequent article on the same subject, in which he stated that in the last two years he had continued to achieve good results, and attributed them less to the large quantity administered than to perseverance in the employment of the remedy. He has found, together with others, that the tincture of lobelia administered in this way will frequently produce vomiting. This effect he attributes to the disagreeable taste of the tincture rather than any specific emetic properties of the drug itself. He was, therefore, led to inaugurate a series of experiments with the active principle of the lobelia—the alkaloid lobeline. Dr. Nunes commenced his experiments with the administration of lobeline in doses of $\frac{1}{6}$ of a grain, given at long intervals, while all other treatment was suspended, and gradually increased the quanity given until 6 grains were given to asthmatics without producing any toxic effects. The tolerance for this large dose of lobeline, claimed to exist by the author in asthmatics, is attributed by him to the excited condition of the nervous system, in which he finds the primary cause of the asthmatic affections.

Lobeline, according to Dr. Nunes, may be administered subcutaneously without producing any local reaction, and he advises its use in this manner where an immediate effect is desired. The author publishes tolerably full notes of nine cases of asthma in which lobeline, in doses varying from ¾ of a grain to six grains, seemed to produce decided relief from the asthmatic symptoms, and he terminates his memoir with the following conclusions: 1. Lobeline does not possess the toxic effects generally attributed to it in the doses which he recommends. 2. It possesses no emetic or nauseating properties, as is the case in lobelia, and its employment is, therefore, preferable where lobelia is indicated. 3. He has employed it in doses of from ¾ of a grain to 6 grains daily for adults; for children, ⅙ to ¾ of a grain. 4. It has no irritating action on the cellular tissue, and therefore may be administered in hypodermic injection—a fact which renders it more preferable than the tincture of lobelia. 5. The evident action of lobeline on the nervous system would seem to indicate its employment in other convulsive affections, such as tetanus. 6. The cases in which he has employed lobeline have remained permanently cured.—*Therapeutic Gazette.*

**Chronic Diarrhœa.**—Dr. Debove claims great success with salicylic of magnesium administered in doses of half an ounce to an ounce and a half a day, suspended in a quart of milk. As a result the diarrhœa disappears.—*Southern Clinic.*

**Injections of Lemon Juice in epistaxis.**—Dr. Geneuil uses an ordinary glass urethral syringe with which he washes out the nostrils with warm water as a preliminary to injecting a syringeful of freshly expressed lemon juice. He has succeeded in arresting promptly several violent attacks of epistaxis by this means, and seldom more than one application was required. The author does not credit the citric acid with the styptic properties of the juice, he having on several occasions substituted a concentrated solution of citric acid without obtaining equally good results. The juice contains other substances besides the acid, to which its effects are probably largely due.—*Ib.*

**Flatulent Dyspepsia.**—Flatulency, or simple eructations of tasteless gas, after eating or drinking the least quantity is a terrible *bore* to many people. Everything they eat or drink seems to create gas in the stomach—all is *ventus et pretarea nihil.* Well here are three prescriptions with a liitle advice that may help the tried physicians' patience and the more tried patients' patience, who has been swallowing drugs *ad infinitum* with no relief—he or she still *eructs.*

Flatus generally results from the excessive formation of gas; then let us try sulphurous acid, strychnia or nux, etc.

℞ Acid sulphurosi, one to two drs. fl. ext. or tr. nucis vom., one dr. tr. cardam, comp., ½ oz. Water, q. s. ad., four oz.

M. Sig; One teaspoonful in water after meals.

Or in *atonic* cases—and these cases may generally be *atonic*, and a local stimulant to the stomach is needed with an antifermentative and antiseptic agent; then *creosote* is an admirable remedy. Give ½ to one hour after meals. It may be combined with bicarb. soda or subnitrate of bismuth, somewhat after this formula:

℞ Creosote, qtt x.
Bism. subnit or sub. carb. dr. ii.
Mucil. acaciae, five oz.

M. Sig: Mix well and give two teaspoonfuls about one hour after meals.

Pepsin or lactopeptine may also be required—any physician can combine either of these articles with creosote, bismuth etc., to meet the case or his views.

Powdered charcoal with soda or bismuth, or magnesia, rhubarb and a little ginger or capsicum often act well, temporarily at least, till the stomach is toned up by other agents; a few drops of oil of cajuput in sugar often relieves.—*Med. Brief.*

**Glycerin in Dyspepsia.**—Gentlemen:—In the *Medical Age* of February 11, 1889, I attempted to call the attention of the medical profession to the very great value of glycerin as a remedy in indigestion and dyspepsia. In addition to what I there said, I would like to make the claim in your journal that glycerin in drachm doses will be found most valuable in preventing stomach troubles in convalescence from debilitating diseases, that it will often cut short an attack of indigestion, and that it will *prevent* and *cure* a large proportion of cases of "summer diarrhœa" of children. It will also to a great extent control the vomiting of pregnancy.

I am most anxious to have these claims thoroughly tested. I am aware that new methods often appear to yield far better results in the hands of the originators than with those who subsequently use them. Therefore I request that every physician having cases of stomach weakness, acute or chronic, from whatever cause, summer diarrhœa, vomiting of pregnancy, etc., test this remedy and report results. It is simple, it is harmless, it is cheap, and it is efficient.—J. A. Pollard, A. M., M. D., in *Therapeutic Gazette.*

**Arteries of the ox as Drainage tubes.**—Dr. Stephen H. Weeks, of Portland, Me., described before the American Surgical Association a new form of absorbable drainage-tube prepared from the arteries of animals. The arteries used are those of the ox. They are separated from their sheaths and cut into tubes four or five inches long. They are then boiled in water for about five minutes. This sterilizes them and hardens their coats. Holes are next cut in their sides, and they are passed over glass rods of different sizes according to the size of the tube desired. They are now placed in corrosive sublimate solution, one to one hundred, and allowed to remain ten minutes. Then they are placed in alcohol, ninety-five per cent, and at the end of twenty-four or forty-eight hours the glass rods are removed, the tubes being kept in alcohol until needed. These tubes are unirritating to the tissues; they are absorbed in from five to seven days and drain the wound perfectly.—*Practice.*

**The Best Antipyretic in Febrile Tuberculosis.**—Prof. Jaccound, of Paris, is credited with the statement that the best antipyretic in febrile tuberculosis is the salicylate of soda. It should be given at a maximum dose of thirty grains in twenty-four hours. At a daily dose of fifteen grains the medicament may be advantageously continued for a long time, with this precaution that the patient should absorb, after each dose or part of a dose, largely diluted with plain or alcoholized water. The contra-indications of the use of the salicylate of soda are: affections of the kidneys, inflammation of the lungs (for fear of asphyxia), and of the heart.—*Ib.*

**Yellow Fever and its Prevention.**—Dr. Nelson predicts that inoculation for yellow fever is destined to take equal rank with that for small-pox, and believes that a new era is at hand in the treatment of this terrible scourge. With reference to its pathology, he looks upon it as a blood disease pure and simple, and that except it be the destruction of blood corpuscles there is often an absence of any other marked pathological evidence, death resulting from necræmia. With such prophylactic treatment and a proper quarantine surveillance, the travel and the commerce of the nations, he believes, will be subject to little interruption. When the opportunity permits we shall be glad to place the entire paper before our readers.—*The Journal.*

**Antidote to Morphine Poisoning.**—Picrotoxin, according to Prof. Bokai, is an antidote to morphine poisoning, acting as a stimulant to the respiratory center, and can be given with more freedom than atropine.—*Med. Brief.*

**Chronic Sore Legs.**—While in the U. S. Navy, during the late civil war, I sent many a sailor to the hospital to be treated for sore legs, while, with my present experience, I would not send one.

In cases of indolent ulcers, non-specific in character, I have poulticed for about twenty-four hours, to clean up the debris, after which I apply, in powder, the following:

| | | |
|---|---|---|
| ℞ | Hyd. chlor. mite | 1 drachm. |
| | Sulph. Quinæ | 1 drachm. |
| | Powd. starch | ½ ounce. |

M. Sig.: Sprinkle parts well.

If patient can remain in bed, no bandage is applied; if patient must be on duty, apply bandage, and let it remain for two days at a time. When cleaned, I only have removed with plain cold water any matter that may present itself; after drying the parts, have them powdered, and repeat the cleansing every two days. I usually succeed in healing the ulcers in about ten days or two weeks. If they are a little tardy in healing, I add one drachm subnit. bismuth.

If I find I have very indolent ulcers, instead of using the poultices or powders, I use, for the first three or four days, the following:

| | | |
|---|---|---|
| ℞ | Cupri sulph. | 1 scruple. |
| | Ung stramoniæ | 1 ounce. |

M. Sig.: Apply two or three time daily.

If I find I have very painful and inflamed ulcers, I first use:

| | | |
|---|---|---|
| ℞ | Hyd. cocaine | 10 grains. |
| | Acid boracic | 2 scruples. |
| | Ung aquæ rosæ | 1 ounce. |

M. Sig.: Apply as required.

After which I use my powder. The last unguent I have always found to act nicely in local imflammations or hyperæsthesia.—A. Trego Shertzer, M. D., in *Med. Brief.*

**A Solvent for Diphtheric Membrane.**—Dr. William C. Wile, in *N. E. Med. Mo.*, says: The following case will illustrate the value of a new solvent for diphtheric membrane which I fear is not fully known to the profession.

James B., an American, 20 years old, was taken with diphtheria on the 10th day of May of the current year. When I first saw him I found all of the characteristic symptoms of this grave disease. Both tonsils were covered with a tough grayish mass so pathognomonic of diphtheria with all of the constitutional symptoms well marked. I put him on the bi-chloride of mercury, one twenty-fourth of a grain every three hours, gave a brisk cathartic and locally ordered a gargle composed of equal parts of Sulphocalcine and water to be used every hour. The next morning when I saw him there was but a thin coating over the tonsils and all of the symptoms were better. On the morning of the eleventh there was a still further improvement and the membrane was all gone. The bi-chloride was given at longer intervals and everything went along nicely, the gargle being used only two or three times a day, and then simply as a prophylactic. On the fifteenth he took a cold and I was sent for hurriedly in the evening. I found that both tonsils, uvulva, pharynx and the whole vault of the mouth was completely covered with a thick diphtheric deposit. I have never seen a case of diphtheria which has relapsed get well, so I gave a very unfavorable prognosis deeming it an impossibility for the patient to recover, with such extensive deposit present. I, however, increased the bi-chloride to every two hours and commenced painting the membrane over with a camel's hair brush every fifteen minutes with an undiluted solution of sulphocalcine, beside having him use the half and half gargle every half hour.

The result was simply marvelous. The membrane commenced to fade and in twenty-four hours there was scarcely a vestige left and recovery was assured. While the patient made a slow and tedious recovery, he did recover and is well and hearty to-day. I regard this one of the most remarkable cases of my experience and I believe that sulphocalcine saved his life.

**Typhoid Fever from Drinking Water.**—That typhoid fever is spread by means of water, is a fact of which there are numerous well authenticated illustrations. Another is contributed by the report of the Board of Health of Providence, R. I., for 1888. This describes an epidemic of typhoid fever extending through the months of November and December of that year. In searching for the cause of this epidemic, the water supply of Providence was investigated. It was found that typhoid fever prevailed at Natick, a village three and one-half miles above the pumping station of the city water-works, which discharged its sewage into the Pawtucket river. An examination of the water direct, failed to reveal the particular organism of typhoid fever, when the water filters of the typhoid fever patients were examined; care was taken to examine only such filters as had no source of contamination other than the river water. The examinations were made by well known experts in this sort of work, and the results showed that not only typhoid baccilli existed in these filters, but several organisms characteristic of fæcal matter.

It would thus appear that the Providence epidemic was due directly to the typhoid baccilli discharged into its drinking water by the fever patients of Natick.

Incidentally, the value of ordinary domestic filters in catching and retaining the typhoid baccillus is made evident. But for these filters the baccilli would in this instance have escaped detection. It is a question whether it were more advantageous to the persons drinking the water from such filters that these baccilli be caught in the filters, or at once carried into the alimentary canal. If the filters could be cleansed by, or in boiling water, daily, it would seem as if the protection would be complete; but as a matter of fact, few if any are thus purified, and so the bacilli multiply in the filter and escape into the filtered water indefinitely. Filters of water are demonstrably of value only to remove course materials held in suspension; the chemical and bacteriological elements which constitute the real sources of danger in drinking water are not usually eliminated. The appearance of muddy water is materially improved by passing through any good filter, and if it be previously boiled it can be said to be safe as well as pleasant to the eye. Dependence upon simple filtration to purify drinking water from the elements of disease, is shown to be a broken reed.—*American Lancet.*

**One Form of Vertigo.**—We often meet with cases of simple vertigo or giddiness, of more or less severity, of short or long continuance, and apparently unconnected with brain disease, dyspepsia, arterial changes, menstruation, hysteria, epilepsy, enemia, congestion, or ear disease. Possibly it is an affection of the cerebellum, or its adjacent large ganglia. The usual remedies for dyspepsia do not relieve it, nor those applicable to the other conditions or diseases above mentioned; but great benefit is usually derived from Turkish baths, change of scene, galvanism, and the internal administration of strychnine.—*Medical World.*

**Treatment of Felons and Gouty Inflammation.**—Moisten the skin around the inflamed part, and rub nitrate of silver stick over it. This is the remedy given by Dr. Gaucher, in the *Journal of the*

*A. M. A.*, by which he says the pain and inflammation are soon subdued.—*Ib.*

**Simple Treatment for Acute Coryza.**—The Schweizer Wochenscrift fur Pharmacie, No. 49, gives the following simple treatment for this affection :

Put one teaspoonful of powdered camphor in a cone-shaped vessel, fill with boiling water, and cover with a cornucopia, the top of which is then torn off just enough to admit the nose, and the warm camphor-vapor inhaled for ten or fifteen minutes. A repetition of the procedure after four or five hours, will generally suffice to effect a cure.—*Correspondence Blatt. f. Schweizer Aerzte.*

**The Treatment of Hæmoptysis.**—Dr. Bottrick, of Hagen, finds opium, with acetate of lead, the most efficacious when bleeding from the lungs has just set in. It combines the action of hæmoptysis and diminution of the cough. After two or three days he gave instead hydrastis, which drug he ordered from the beginning when the hemorrhage was only slight. It had mostly a very decided effect. In one case of hysterical hæmoptysis its administration entirely stopped hemorrhage after the first week. Also in cases of frequent bleeding from the nose, hydrastis proved very serviceable, not against a single attack of profuse epistaxis, but against repeated recurrence of the bleeding. In such cases he has used the drug for the last two years, and is very well satisfied with the results.—*N. Y. Med. Record.*

**Antifebrin.**—A thirty-eight year old book-binder received two doses of antifebrin of thirty grains each in the course of a day. After the second dose the following symptoms appeared: Cool sweat, tiredness, vertigo, confusion of mind, anxiety, palpitation of the heart, small pulse, and intense cyanosis. These symptoms gradually disappeared, although the weakness persisted several days.—*Allg. Hom. Zeitung*, No. 20, 1889.

**Poisoning by Glycerine.**—A patient suffering from diabetes' took, by the advice of a friend, large quantities of glycerine, both by the mouth and by the anus. He purchased a cheap grade of the drug. After two weeks' use, gastric and abdominal symptoms set in, and when medical aid was applied for, he had a perfect picture of cholera nostral; collapse, vomiting, painful diarrhœa, cramps in the calves of the legs, etc., all of which ceased under suitable treatment, and discontinuance of the glycerine. It is a well-known fact that the glycerine of the trade contains considerable arsenic, and to this the attack above described may be attributed.—*Weiner Med. Presse*, 23, 1889.

**Removal of Warts.**—The skin surrounding the wart should be protected with cotton. Then apply the liquid carbolic acid to the wart, and allow it to dry. No pain is perceptible. In the course of two or three days a part of the wart will fall off. Renew the application until all has been removed.—*Waif.*

